# Estimated Cardiac Risk Associated With Macrolides and Fluoroquinolones Decreases Substantially When Adjusting for Patient Characteristics and Comorbidities

**DOI:** 10.1161/JAHA.117.008074

**Published:** 2018-04-21

**Authors:** Linnea A. Polgreen, Benjamin N. Riedle, Joseph E. Cavanaugh, Saket Girotra, Barry London, Mary C. Schroeder, Philip M. Polgreen

**Affiliations:** ^1^ Department of Pharmacy Practice and Science University of Iowa Iowa City IA; ^2^ Department of Biostatistics University of Iowa Iowa City IA; ^3^ Department of Internal Medicine University of Iowa Iowa City IA; ^4^ Department of Molecular Physiology and Biophysics University of Iowa Iowa City IA; ^5^ Department of Epidemiology University of Iowa Iowa City IA

**Keywords:** cardiac, medication, morbidity, mortality, Cardiovascular Disease, Epidemiology, Myocardial Infarction

## Abstract

**Background:**

Some studies have found that antimicrobials, especially macrolides, increase the risk of cardiovascular death. We investigated potential cardiac‐related events associated with antimicrobial use in a population of patients with acute myocardial infarction.

**Methods and Results:**

For 185 010 Medicare beneficiaries, we recorded prescriptions for azithromycin, clarithromycin, levofloxacin, moxifloxacin, doxycycline, and amoxicillin‐clavulanate. In the following week, we recorded death, acute myocardial infarction, atrial fibrillation or atrial flutter, a non–atrial fibrillation/atrial flutter arrhythmia, or ventricular arrhythmia. We fit unadjusted and adjusted logistic regression models using generalized estimating equations. Adjusted models included patients’ comorbidities, medications, procedures, demographics, insurance status, time since index acute myocardial infarction, number of visits, and the influenza rate. In unadjusted analyses, macrolides and fluoroquinolones were associated with a risk of cardiac events. However, the risk associated with macrolide use was substantially attenuated after adjustment for a wide range of variables, and the risk associated with fluoroquinolones was no longer statistically significant. For example, for azithromycin, the odds ratio for any cardiac event or death was 1.35 (95% confidence interval, 1.27–1.44; *P*<0.0001), but after controlling for a wide range of covariates, the odds ratio decreased to 1.01 (95% confidence interval, 0.95–1.08; *P*<0.6688).

**Conclusions:**

Controlling for covariates explains much of the adverse cardiac risk associated with antimicrobial use found in other studies. Most antimicrobials are not associated with risk of cardiac events, and others, specifically azithromycin and clarithromycin, may pose a small risk of certain cardiac events. However, the modest potential risks attributable to these antimicrobials must be weighed against the drugs’ considerable and immediate benefits.

**Clinical Perspective**

**What Is New?**

- We examine the effects of antimicrobials on cardiac outcomes because many antimicrobials, especially macrolides and fluoroquinolones, have been associated with adverse cardiac outcomes, including death.
- In this study, we found that the increased risk in cardiovascular outcomes associated with antimicrobials is largely explained by underlying patient factors and the indication for which the antimicrobial was used, rather than directly because of the antimicrobial agent itself.

**What Are the Clinical Implications?**

- Macrolides and fluoroquinolones are effective antimicrobials used to treat serious respiratory infections and play an important role in the treatment of community‐acquired pneumonia.
- The modest potential risks attributable to these antimicrobials must be weighed against the drugs’ considerable and immediate benefits.

## Introduction

Antimicrobial agents are commonly prescribed for treating infections. However, recent studies have raised concerns about the risk of cardiovascular events associated with their use. Macrolides are commonly used to treat respiratory tract infections, and their mechanism of action against respiratory pathogens is to inhibit bacterial protein synthesis. Macrolide use is also associated with QT prolongation, and multiple case reports describe cardiac arrhythmias in patients treated with macrolides.^1^, ^2^, ^3^, ^4^, ^5^, ^6^, ^7^ However, despite these reports, multiple large retrospective clinical studies investigating the potential risk of the use of macrolides in practice have yielded conflicting results. In some studies, azithromycin, for example, has been associated with negative cardiac outcomes and death.^8^, ^9^, ^10^, ^11^, ^12^, ^13^, ^14^ Specifically, Ray et al found that in a Medicaid cohort, patients prescribed azithromycin had an increased risk of death compared with patients prescribed amoxicillin.^8^ In another study, Chou et al found cardiac risk not only for macrolides, but also for fluoroquinolones, when compared with amoxicillin‐clavulanate use.^13^ In contrast, Svanstrom et al demonstrated no increased risk of death from cardiovascular causes from azithromycin in a large population of middle‐aged adults in Denmark, compared with penicillin V,^10^ and Trifiro et al found no risk of ventricular arrhythmia (VA) from azithromycin when compared with amoxicillin.^15^

The divergent findings noted in the previously described studies may be attributable to differences in study populations. The increased risk associated with azithromycin has been most prominent in studies that included high‐risk patients (eg, patients already experiencing cardiovascular diseases).^10^, ^13^ Moreover, the extent to which adverse cardiovascular effects of antimicrobials are attributable to selection bias (confounding by indication) remains unknown. For example, pneumonia, a common indication for macrolides, also increases the risk of cardiac arrhythmias and death. In contrast, amoxicillin is usually prescribed for milder infections (eg, ear or tooth infections). Therefore, there is potential for confounding because of indication in studies that compare cardiovascular outcomes in patients prescribed macrolides with patients prescribed amoxicillin or penicillin. In addition, the results of many of these studies may be attributable to omitted variable bias: failing to fully account for both the indications for the antimicrobials and the characteristics of the patients receiving them is likely to lead to the antimicrobials appearing riskier than they actually are.

Given the conflicting findings of prior investigations, the purpose of this study was to investigate the cardiac risk associated with treatment with macrolides, fluoroquinolones, and other antimicrobials in a high‐risk population (ie, a large cohort of people who have previously experienced an acute myocardial infarction [AMI]), controlling for a variety of patient and environmental variables not included in previous studies. We specifically hypothesize that, after adjusting for important covariates, the studied antimicrobials will not have a clinically meaningful or statistically significant effect on the studied outcomes.

## Methods

On request, the code for executing the analytic methods used in this article will be made available by the corresponding author. L.A.P. has full access to all the data in the study and is responsible for their integrity and the data analysis. Because of data use agreements, the data will not be made available.

### Data Sources

For the present study's analyses, we used Medicare Parts A and B claims and enrollment information as well as part D prescription drug events from the Chronic Condition Data Warehouse (http://www.ccwdata.org). Medicare Part A is hospital insurance, which includes care received in a hospital or at a skilled nursing facility. Medicare Part B is medical insurance that covers medically necessary physicians’ services, durable medical equipment, and outpatient services. Medicare Part D provides outpatient prescription drug coverage. We also used information from the US Census to help quantify a patient's socioeconomic status.

### Study Cohort

In this study, we identified all Medicare beneficiaries aged ≥66 years, who were discharged alive from a hospital with a diagnosis of AMI (*International Classification of Diseases, Ninth Revision* [*ICD‐9*] code 410.x1) between January 1, 2007, and December 31, 2008. The date of admission for the inpatient stay in which the AMI occurred served as the index admission date, and the index discharge date was the date in which the patient was discharged home. Further criteria for inclusion in the study were as follows: (1) having complete claims information within the 12 months before index admission; (2) not having had an AMI within the 12 months before index admission; (3) being enrolled in Medicare Parts A and B during the 12 months before index admission; (4) being enrolled in Medicare Part D during the 6 months before index admission; and (5) having complete claims information and being enrolled in Medicare Parts A, B, and D until either the patient's death or 12 months after index discharge, whichever occurred first. This study was approved by the institutional review board of the University of Iowa (Iowa City, IA). Informed consent was waived for this study.

### Study Antimicrobials

Starting from a patient's index discharge date, we divided the following year into “patient‐weeks.” Specifically, for a given patient, patient‐week 1 is days 1 through 7 after discharge; week 2 is days 8 through 14 after discharge, etc. In the event a patient died within the year after his or her index date, any patient‐week in which the patient was already deceased at the start of the week was not included in any analyses. For each patient‐week, Medicare Prescription Drug Event files were used to determine whether a patient had filled a prescription for any of the following antimicrobials of interest: azithromycin, clarithromycin, levofloxacin, moxifloxacin, doxycycline, and amoxicillin‐clavulanate. Our choice of the specific antimicrobials was based on prior literature suggesting that macrolides, and possibly fluoroquinolones, are associated with cardiac events.^8^, ^13^

### Defining Outcomes and Predictors

In each patient‐week, we determined whether a patient died or had a claim for one of the following cardiac events: AMI, stroke (any claim with *ICD‐9* code 430, 431, 434.00, 434.01, 434.10, 434.11, 434.90, 434.91, 435.0, 435.1, 435.3, 435.8, 435.9, 436, or 997.02), transient ischemic attack (*ICD‐9* code 435.x), unstable angina (*ICD‐9* code 411.xx), atrial fibrillation (AFI; *ICD‐9* code 427.31), atrial flutter (AFL; *ICD‐9* code 427.32), any cardiac arrhythmia (*ICD‐9* code 427.xx or 798.xx), and VA (*ICD‐9* code 427.1, 427.4, 427.41, or 427.42), which is a subset of cardiac arrhythmia.

In this article, we consider 6 outcomes: death, AMI, VA, a combination of AFI and AFL, any non‐AFI/AFL arrhythmia (defined as any cardiac arrhythmia [*ICD‐9* code 427.xx or 798.xx], except for AFI [*ICD‐9* code 427.31] and AFL [*ICD‐9* code 427.32]), and any event (defined as death or any cardiac event previously listed). Patients experiencing a VA by definition also experience a non‐AFI/AFL arrhythmia. Also, because most patients will have multiple claims with multiple diagnoses, then patients may experience many or all of these 6 outcomes.

Because many patient attributes may confound the relationship between antimicrobials and cardiac events, we control for >100 patient covariates. Some of the covariates for which we controlled were the following: national rate of influenza‐like illness in the given week, because influenza is often inappropriately treated with antimicrobials and is also associated with cardiac events^16^; a count of a patient's total inpatient and outpatient visits in the given week and previous week, which approximately captures the patient's health in the given week; indicators for common indications for antimicrobials (pneumonia, urinary tract infection, and chronic obstructive pulmonary disease) in the previous or given week; indicators of whether the given week occurred in the first, second, or third month after the index AMI date; patient demographics, including age, sex, and race; preindex and at‐index comorbidities, including stroke, heart failure, cardiac arrest, hypotension, hypertension, renal failure, chronic kidney disease, diabetes mellitus, cancer, pneumonia, depression, asthma, and hyperkalemia; procedures during the AMI stay, including pacemaker implant, cardiac catheterization, percutaneous coronary intervention, and coronary artery bypass grafting; Medicare Part D insurance variables, including premium levels, Medicare benefit phase, and beneficiary accumulated total and out‐of‐pocket drug costs; whether patients were Medicaid eligible; whether patients received a low‐income subsidy; and other zip code–level socioeconomic characteristics from the US Census, such as per capita income, poverty rate, education level, English‐speaking percentage, and life expectancy. We account for whether the week occurred in one of the first 3 months after discharge, because patients were considerably more likely to experience cardiac events in the months immediately after discharge. The same control variables were included in all of the final models. These control variables were determined by applying a backwards elimination model selection procedure to a full model formulated for each of the studied outcomes. A variable was included in all of the final models if it was selected for at least one of the outcomes.

### Statistical Analyses

We begin by providing a summary of the sex, race, age, measures of socioeconomic status, and 1‐year outcomes for the full cohort, as well as for the cohort of antimicrobial users and antimicrobial nonusers. Patients were defined as antimicrobial users if and only if they filled a prescription for at least one of the studied antimicrobials. The *P* value from a χ^2^ test of independence between antimicrobial use status and the given variable is provided. These results are presented in Table 1.

**Table 1:** The Sex, Race, Age, Socioeconomic, and Health Characteristics of the Full Study Cohort, the Antimicrobial Users (Defined as Any Patient Who Filled at Least 1 Prescription for 1 of the Studied Antimicrobials in the Year After Index) and Antimicrobial Nonusers

Characteristic	Full Cohort, Count (%)	Antimicrobial Users, Count (%)	Antimicrobial Nonusers, Count (%)	*P* Value Comparing Antimicrobial Users With Nonusers
Total patients	185 010	76 940	108 070	…
Male sex	75 073 (40.58)	30 353 (39.45)	44 720 (41.38)	<0.0001
Race				
White	153 242 (82.83)	63 983 (83.16)	89 259 (82.59)	0.0015
Black	15 412 (8.33)	5647 (7.34)	9765 (9.04)	<0.0001
Hispanic	10 781 (5.83)	4946 (6.43)	5835 (5.40)	<0.0001
Age, y				
66–70	33 571 (18.15)	14 589 (18.96)	18 982 (17.56)	<0.0001
71–75	34 313 (18.55)	14 941 (19.42)	19 372 (17.93)	<0.0001
76–80	37 238 (20.13)	15 730 (20.44)	21 508 (19.90)	0.0041
81–85	36 002 (19.46)	14 633 (19.02)	21 369 (19.77)	<0.0001
≥85	43 886 (23.72)	17 047 (22.16)	26 839 (24.83)	<0.0001
Socioeconomic factors				
Receives low‐income subsidy	11 072 (5.98)	4584 (5.96)	6488 (6.00)	0.6834
High no‐English area	52 319 (28.28)	21 726 (28.24)	30 593 (28.31)	0.7385
Dually eligible for Medicaid	68 498 (37.02)	30 907 (40.17)	37 591 (34.78)	<0.0001
Measures of 1‐y postindex health				
Any event	120 707 (65.24)	50 928 (66.19)	69 779 (64.57)	<0.0001
Death	43 645 (23.59)	14 450 (18.78)	29 195 (27.01)	<0.0001
Acute myocardial infarction	18 247 (9.86)	8368 (10.88)	9879 (9.14)	<0.0001
Non‐AFI/AFL arrhythmia	68 145 (36.83)	30 448 (39.57)	37 697 (34.88)	<0.0001
Ventricular arrhythmia	13 974 (7.55)	6321 (8.22)	7653 (7.08)	<0.0001
AFI/AFL	62 856 (33.97)	27 892 (36.25)	34 964 (32.35)	<0.0001

The measures of health counts represent the number of patients who experienced the given outcome at least once in the year after index. AFI indicates atrial fibrillation; and AFL, atrial flutter.

For all further statistical analyses, the unit of analysis was the patient‐week. The dependent variable was an indicator reflecting whether, in each patient‐week, the patient had a claim for the considered outcome. The primary independent variable of interest was an indicator denoting whether the patient had filled a prescription for a particular antimicrobial previously listed *in the previous week*. Specifically, we are comparing patient‐weeks in which the given antimicrobial was prescribed with those in which it was not. By looking back 1 week to determine if an antimicrobial prescription was filled, we ensure that, in any instance in which both a cardiac event occurred and an antimicrobial prescription was filled, the prescription was filled before the cardiac event occurred. We do not include in the analyses each patient's week immediately after discharge because we cannot look back to the previous week to determine if an antimicrobial was prescribed. Thus, each patient's first entry is whether he or she experienced the given outcome in week 2 after index and received an antimicrobial prescription in week 1. Any patient who lived the entire year after index will contribute 51 separate entries for analysis.

For both the adjusted and unadjusted analyses, we accounted for within‐person correlation by fitting logistic regression models using generalized estimating equations. An independent working correlation structure was used, and robust standard errors were computed for the regression parameter estimates. Thus, the models were fit using the quasi‐likelihood approach. Models were fit for each combination of cardiac outcome and antimicrobial. Confidence intervals (CIs) and tests were based on the Wald approach.

The unadjusted analyses use only whether the patient was prescribed the given antimicrobial in the previous week as a predictor. The adjusted analyses include the antimicrobial predictor and all control covariates listed in the previous section. For each of the 6 antimicrobials, we consider the effect of receiving the particular antimicrobial on each of the outcomes. We also consider the effect of receiving any of the antimicrobials. Thus, because we consider a total of 7 predictors of interest and 6 outcomes, we fit 42 unadjusted and 42 adjusted models. For each combination of outcome and antimicrobial, we calculated both an adjusted and unadjusted odds ratio (OR), as well as a corresponding 95% CI and *P* value for each.

Because we are presenting a considerable number of results from the adjusted and unadjusted analyses, applying a correction for multiple comparisons to assess statistical significance is prudent. We thus applied a Bonferroni correction, which, on the basis of performing 42 comparisons, requires a *P*≤0.05/42=0.0012 to achieve statistical significance.

Finally, the reference group in our primary analyses is patient‐weeks without a prescription for the given antimicrobial; thus, the reference group will contain patient‐weeks in which other antimicrobials are used. We, therefore, perform a sensitivity analysis in which, for each studied antimicrobial, only patients who were not prescribed one of the other studied antimicrobials were included in the cohort. Thus, for each antimicrobial, the baseline will be patient‐weeks in which none of the studied antimicrobials was prescribed. We performed these adjusted and unadjusted sensitivity analyses for each combination of the 7 antimicrobials and 6 outcomes. All analyses were performed using SAS 9.4.

## Results

A total of 8 091 008 patient‐weeks were used in the adjusted and unadjusted analyses. In our cohort of 185 010 patients, 18 247 (9.86%) experienced a second AMI in the year after their index date. Because 1845 AMIs occurred in the first week after the index date, the analyses used 16 402 patient‐weeks in which an AMI occurred. Similarly, the number of analyzed patient‐weeks in which the given outcome occurred were as follows: 150 258 cardiac events or deaths, 43 134 deaths, 65 637 non‐AFI/AFL arrhythmias, 12 855 VAs, and 47 336 AFIs/AFLs. A total of 9934 moxifloxacin prescriptions were filled for patients in the year after their index date; there were 18 683 amoxicillin‐clavulanate prescriptions, 14 677 doxycycline prescriptions, 40 119 azithromycin prescriptions, 54 620 levofloxacin prescriptions, and 3465 clarithromycin prescriptions.

Patient characteristics for the entire cohort, including antimicrobial users and nonusers, are displayed in Table 1. The results for the measures of 1‐year postindex health presented in Table 1 signify the number and proportion of patients who experienced the given outcome at least once in the year after discharge. From Table 1, we see that the overall study cohort was ≈60% women and >80% white, with an approximately uniform distribution of ages between 66 and 85 years, and just <25% were >85 years. Most patients came from a low‐income area, and 37% were dually eligible for Medicaid. More than 75% of patients survived more than a year after their index date, and ≈35% survived and experienced none of the studied cardiac events in the year after their index date. For many of the demographic and socioeconomic factors, the antimicrobial users were similar to the nonusers. However, the antimicrobial users appear to be younger and have a higher chance of being dually eligible for Medicaid on the index date (40.17% versus 34.78%; *P*<0.0001). Antimicrobial users appear to have a higher chance of 1‐year survival (*P*<0.0001), with a 1‐year survival rate of 81.22% compared with 72.99% for nonusers. We should be careful not to overinterpret this result, however. Because patients dying early in the study period will have less time to be prescribed an antimicrobial, surviving patients may be biased towards receiving an antimicrobial simply because they were alive for the entire study period. Table 1 also shows that antimicrobial users are also more likely to experience at least one recurrent AMI, non‐AFI/AFL arrhythmia, VA, and AFI/AFL (all *P*<0.0001) in the year after discharge.

Adjusted (multivariable) and unadjusted (univariable) ORs as well as 95% CIs and *P* values for each combination of antimicrobial and cardiac event are displayed in Table 2.

**Table 2:** The Adjusted and Unadjusted ORs for Each Combination of Antimicrobial and Cardiac Outcome, as Well as Their 95% CIs and *P* Values

Antimicrobial	Event	Unadjusted OR	95% Unadjusted CI	Unadjusted *P* Value	Adjusted OR	95% Adjusted CI	Adjusted *P* Value
Macrolides							
Azithromycin (n=40 119)	Any event (n=150 258)	1.35^a^	(1.27–1.44)	<0.0001	1.01	(0.95–1.08)	0.6688
	Death (n=43 134)	1.07	(0.94–1.22)	0.3211	0.74^a^	(0.65–0.85)	<0.0001
	Acute myocardial infarction (n=16 402)	1.47^a^	(1.23–1.77)	<0.0001	1.10	(0.91–1.33)	0.3052
	Non‐AFI/AFL arrhythmia (n=65 637)	1.27^a^	(1.15–1.40)	<0.0001	1.02	(0.93–1.13)	0.6472
	Ventricular arrhythmia (n=12 855)	1.41	(1.14–1.73)	0.0013	1.13	(0.92–1.40)	0.2450
	AFI/AFL (n=47 336)	1.63^a^	(1.47–1.81)	<0.0001	1.24^a^	(1.11–1.38)	0.0001
Clarithromycin (n=3465)	Any event (n=150 258)	1.68^a^	(1.38–2.03)	<0.0001	1.24	(1.00–1.52)	0.0454
	Death (n=43 134)	1.24	(0.82–1.87)	0.3036	0.92	(0.60–1.42)	0.7173
	Acute myocardial infarction (n=16 402)	2.12	(1.28–3.52)	0.0037	1.49	(0.86–2.58)	0.1558
	Non‐AFI/AFL arrhythmia (n=65 637)	1.64^a^	(1.22–2.19)	0.0009	1.19	(0.87–1.61)	0.2735
	Ventricular arrhythmia (n=12 855)	1.63	(0.85–3.13)	0.1448	1.19	(0.62–2.28)	0.6108
	AFI/AFL (n=47 336)	2.35^a^	(1.77–3.11)	<0.0001	1.70^a^	(1.23–2.33)	0.0011
Fluoroquinolones							
Levofloxacin (n=54 620)	Any event (n=150 258)	2.18^a^	(2.09–2.28)	<0.0001	0.87^a^	(0.83–0.91)	<0.0001
	Death (n=43 134)	2.15^a^	(1.98–2.32)	<0.0001	0.82^a^	(0.75–0.89)	<0.0001
	Acute myocardial infarction (n=16 402)	2.05^a^	(1.80–2.34)	<0.0001	0.88	(0.76–1.01)	0.0660
	Non‐AFI/AFL arrhythmia (n=65 637)	1.98^a^	(1.85–2.12)	<0.0001	0.90	(0.84–0.97)	0.0068
	Ventricular arrhythmia (n=12 855)	1.95^a^	(1.68–2.27)	<0.0001	0.98	(0.84–1.16)	0.8455
	AFI/AFL (n=47 336)	2.41^a^	(2.24–2.59)	<0.0001	0.89	(0.82–0.97)	0.0080
Moxifloxacin (n=9934)	Any event (n=150 258)	2.06^a^	(1.86–2.25)	<0.0001	0.83^a^	(0.74–0.93)	0.0011
	Death (n=43 134)	2.02^a^	(1.67–2.45)	<0.0001	0.74	(0.60–0.90)	0.0032
	Acute myocardial infarction (n=16 402)	1.41	(0.97–2.05)	0.0692	0.57	(0.39–0.85)	0.0058
	Non‐AFI/AFL arrhythmia (n=65 637)	2.07^a^	(1.78–2.42)	<0.0001	1.00	(0.85–1.18)	0.9765
	Ventricular arrhythmia (n=12 855)	1.83^a^	(1.27–2.64)	0.0011	0.82	(0.56–1.22)	0.3346
	AFI/AFL (n=47 336)	2.59^a^	(2.20–3.05)	<0.0001	0.97	(0.81–1.17)	0.7807
Other antimicrobials							
Doxycycline (n=14 677)	Any event (n=150 258)	1.12	(1.00–1.25)	0.0567	0.81^a^	(0.72–0.91)	0.0004
	Death (n=43 134)	1.00	(0.80–1.25)	0.9879	0.69	(0.55–0.87)	0.0014
	Acute myocardial infarction (n=16 402)	1.04	(0.73–1.48)	0.8269	0.81	(0.57–1.16)	0.2496
	Non‐AFI/AFL arrhythmia (n=65 637)	1.16	(0.98–1.37)	0.0902	0.91	(0.76–1.08)	0.2659
	Ventricular arrhythmia (n=12 855)	0.86	(0.55–1.33)	0.5010	0.69	(0.44–1.08)	0.1041
	AFI/AFL (n=47 336)	1.36^a^	(1.13–1.64)	0.0011	0.98	(0.80–1.21)	0.8724
Amoxicillin‐clavulanate (n=18 683)	Any event (n=150 258)	1.76^a^	(1.62–1.91)	<0.0001	0.91	(0.83–0.99)	0.0308
	Death (n=43 134)	1.82^a^	(1.57–2.11)	<0.0001	0.87	(0.75–1.02)	0.0868
	Acute myocardial infarction (n=16 402)	1.64^a^	(1.27–2.11)	0.0001	0.87	(0.67–1.13)	0.3030
	Non‐AFI/AFL arrhythmia (n=65 637)	1.48^a^	(1.30–1.70)	<0.0001	0.86	(0.74–0.98)	0.0295
	Ventricular arrhythmia (n=12 855)	1.40	(1.03–1.90)	0.0336	0.79	(0.58–1.09)	0.1504
	AFI/AFL (n=47 336)	2.02^a^	(1.76–2.32)	<0.0001	1.04	(0.89–1.21)	0.6318
Any studied antimicrobial (n=140 676)	Any event (n=150 258)	1.56	(1.51–1.60)	<0.0001	0.82	(0.79–0.84)	<0.0001
	Death (n=43 134)	1.66	(1.57–1.76)	<0.0001	0.82	(0.77–0.87)	<0.0001
	Acute myocardial infarction (n=16 402)	1.55	(1.41–1.70)	<0.0001	0.81	(0.74–0.90)	<0.0001
	Non‐AFI/AFL arrhythmia (n=65 637)	1.50	(1.43–1.57)	<0.0001	0.90	(0.85–0.94)	<0.0001
	Ventricular arrhythmia (n=12 855)	1.51	(1.36–1.68)	<0.0001	0.92	(0.83–1.03)	0.1687
	AFI/AFL (n=47 336)	1.58	(1.50–1.66)	<0.0001	0.86	(0.81–0.91)	<0.0001

The ORs compare the odds of the given outcome for weeks in which the given antimicrobial was prescribed in the previous week with the odds for weeks in which the given antimicrobial was not prescribed in the previous week. The adjusted ORs adjust for all control covariates listed in the Methods section. AFI indicates atrial fibrillation; AFL, atrial flutter; CI, confidence interval; and OR, odds ratio.

a Denotes a statistically significant result when using a Bonferroni correction, in which *P*≤0.0012 is necessary to reach statistical significance.

For each combination of antimicrobial and outcome, the unadjusted OR estimate was larger than the adjusted OR estimate. For example, in the unadjusted analyses, we estimate that the odds of experiencing any event (defined as death or any of the studied cardiac events) in a week when azithromycin was prescribed in the previous week was 1.35 (95% CI, 1.27–1.44; *P*<0.0001) times that of the odds when azithromycin is not prescribed. However, the adjusted OR for azithromycin was 1.01 (95% CI, 0.95–1.08; *P*<0.6688). Using the adjusted analyses, the only drugs that were associated with a significantly increased risk of any of the 6 studied outcomes were the macrolides (ie, azithromycin and clarithromycin). Specifically, the adjusted OR for azithromycin and clarithromycin for the AFI/AFL outcome were 1.24 (95% CI, 1.11–1.38; *P*=0.0001) and 1.70 (95% CI, 1.23–2.33; *P*=0.0011), respectively. For the predictor that combines all of the studied antimicrobials, the adjusted analyses suggest that receiving an antimicrobial is associated with a reduced risk for each of the studied outcomes, except for VA.

Considering the fluoroquinolones and other nonmacrolide antimicrobials, for the composite outcome that represents any event, the unadjusted ORs for levofloxacin, moxifloxacin, and amoxicillin‐clavulanate are 2.18 (95% CI, 2.09–2.28; *P*<0.0001), 2.06 (95% CI, 1.86–2.25; *P*<0.0001), and 1.76 (95% CI, 1.62–1.91; *P*<0.0001), respectively. However, for the same composite outcome, the adjusted ORs for these 3 antimicrobials are 0.87 (95% CI, 0.83–0.91; *P*<0.0001), 0.83 (95% CI, 0.74–0.93; *P*=0.0011), and 0.91 (95% CI, 0.83–0.99; *P*=0.0308), respectively.

According to the adjusted analyses, none of the 6 studied antimicrobials significantly increased the risk of death. In fact, the risk of death is significantly decreased for azithromycin (OR, 0.74; 95% CI, 0.65–0.85; *P*<0.0001) and levofloxacin (OR, 0.82; 95% CI, 0.75–0.89; *P*<0.0001).

In addition, patients are considerably more likely to experience another event in the months immediately after their index discharge. In results not shown, the adjusted azithromycin model indicates that the odds of experiencing an event in the month after discharge were 3.88 (95% CI, 3.82–3.94; *P*<0.0001) that of the odds in months 4 through 12 after discharge. Similarly, the ORs for months 2 and 3 after discharge were 2.35 (95% CI, 2.32–2.39; *P*<0.0001) and 1.65 (95% CI, 1.62–1.68; *P*<0.0001), respectively.

Finally, in the sensitivity analyses in which the reference group consists of patient‐weeks in which none of the studied antimicrobials were prescribed, our findings were not substantially altered, although the sample size of the reference group was reduced (data not shown).

## Discussion

Our results show that, in a population at high risk for arrhythmias and other cardiac‐associated adverse events, the risk of cardiac events associated with macrolide use is dependent on the extent to which we adjust for comorbidities and patient demographics. For azithromycin, the drug most often considered in cardiac‐risk studies, the OR for any event (defined as any cardiac event or death) among patients who had previously experienced an AMI was 1.35, but when we controlled for a wide range of patient and environmental covariates, the OR decreased to 1.01 (*P*=0.6688), a considerably smaller and statistically insignificant effect, especially considering the population of high‐risk patients we analyzed. We find an even more striking reduction of risk for fluoroquinolones. For instance, for levofloxacin, the unadjusted OR for the composite outcome representing any event was 2.18 versus the adjusted OR of 0.87. The differences in the results based on our unadjusted and adjusted models help explain the conflicting results in the observational studies to date by demonstrating how the associated risk depends on the variables considered. Our adjusted analyses controlled for 108 covariates. All control covariates in our models were chosen for inclusion by a backwards elimination model selection procedure. In results not shown, we began the procedure with 143 variables, and thus eliminated 35 because they were deemed unimportant by the model selection procedure. We were thus able to control for potentially important covariates for which previous studies were unable to control. The dissimilarity between our findings and those of some previous studies, which found that antimicrobials increased the risk of some cardiac events, may be explained by the difference in the covariates for which we controlled.

Given our large cohort, we were able to explore the effects of a wide range of covariates on the estimated risk of macrolides on cardiac risk. In results not shown, we found the fit of the adjusted models to be much better than the fit of the unadjusted models. For instance, for azithromycin and all events, the unadjusted model's Quasi‐Likelihood Information Criterion^17^ was >225 000 units larger than the adjusted model's, indicating overwhelming support for the adjusted model. As we added covariates, the risk associated with the examined antimicrobials decreased. The improved fit of our statistical models could merely be attributable to the addition of more covariates. However, because Quasi‐Likelihood Information Criterion penalizes for model complexity, the Quasi‐Likelihood Information Criterion differences provide strong evidence that the covariates are explaining a substantial degree of variation in the outcomes. Moreover, the inclusion of the composite set of covariates in the final adjusted models not only substantially improved model fit, but also changed the estimated model coefficients, indicating lower risk. Thus, we believe that most of the risk associated with macrolides in other studies is because of omitted variable bias. More important, in the case of fluoroquinolones, after adjusting for all the covariates, we find no increased risk of the cardiac events we investigated.

Patients who receive antimicrobials are different from patients who do not, and when patients receive antimicrobials, something is characteristically different about their state of health. The patient may not have an infection, but he or she has a complaint that requires him or her to seek care, and a prescriber is acknowledging a problem in the form of an antimicrobial prescription. Accordingly, antimicrobials may be a marker for increased cardiac risk. For example, pneumonia, the reason for many macrolide and fluoroquinolone prescriptions, can cause arrhythmias,^18^ and many patients hospitalized for pneumonia also have other cardiac events.^19^, ^20^, ^21^ Furthermore, antimicrobials are frequently inappropriately prescribed for influenza and other respiratory tract viruses,^22^ and influenza is thought to be a risk factor for AMIs^16^, ^23^ as well as chronic obstructive pulmonary disease exacerbations,^16^ which can, in turn, lead to cardiac events.^24^ Indeed, in our unadjusted analysis, both doxycycline and amoxicillin clavulanate, 2 drugs that have not been associated with arrhythmias, were associated with increased risk for cardiac events. Similarly, Chou et al used amoxicillin‐clavulanate as their control group, and they found increased risks for this drug as well.^13^ The number of months after discharge strongly predicted whether a patient will experience another event. For instance, the odds of experiencing an event in the month after discharge for the index AMI were nearly 4 times the odds in months 4 through 12 after discharge, indicating that physicians should closely monitor patients immediately after an AMI. Interestingly, we found that the number of clinic or hospital visits was also predictive of cardiac events, regardless of whether the visit resulted in an antimicrobial prescription or not (data not shown). Thus, in our population, visits in general appear to be a risk factor. Of course, these visits do not cause cardiac events: they are most likely a marker of some underlying illness. The reason for the visit, not the visit itself, is the risk factor for unfavorable outcomes.

Conversely, our adjusted analyses showed that the studied antimicrobials were not associated with a statistically significant increase in the risk of any‐cause death; in fact, azithromycin, levofloxacin, moxifloxacin, and doxycycline were associated with a significant decrease in the risk of death. When patients receive antimicrobials, they may be sicker than their baseline health status, but healthier patients may be more apt to pursue antimicrobial prescriptions than their extremely sick counterparts. This could help explain the significant decrease in the risk of death associated with these 4 antimicrobials; patients willing to pursue antimicrobial prescriptions are well enough that their risk of death in the following week is relatively small. This result may also be partially explained by the behavior of the healthcare provider. If a patient appears to be likely to die in the near future, the provider may be more likely to choose more extreme medical care, without prescribing an antimicrobial, even when appropriate.

The relatively small risks we identified for azithromycin among patients who had a previous AMI are consistent with the findings of previous randomized controlled trials using azithromycin in patients with a history of cardiac disease. In 2 large randomized controlled trials,^25^, ^26^ patients were given macrolides for extended periods of time with the thought that they might improve cardiac outcomes by treating subclinical chlamydial infections. Although known adverse events, such as gastrointestinal distress, were reported, no statistically significant cardiac events were observed. It is possible that the randomized trials were not sufficiently powered to detect adverse cardiac events, but nevertheless, both trials were large (4012 and 7747 patients), and treatment with azithromycin continued for a long time (1 year and 3 months) with no significant increases in adverse cardiac events reported in the intervention group compared with controls.^25^, ^26^

Although adjusting for important covariates produced a marked reduction in risk associated with macrolide use, some statistically significant risk remains, and providers must take the patient's clinical situation into account. After adjustment, the largest OR for the composite outcome representing any event was 1.24 for clarithromycin. Despite these risks being modest, we cannot dismiss the possibility that these antimicrobials are associated with some increase in cardiac risk. This relatively small, but statistically significant, risk provides yet one more rationale for avoiding inappropriate antimicrobial prescribing. Unfortunately, inappropriate prescribing for antimicrobials is extremely common.^22^ Thus, even if the risk is small, given the large number of inappropriate antimicrobial prescriptions, it is likely that some adverse cardiac‐associated events occur.

Our study is subject to several limitations. First, our cohort is composed of only patients with AMI, and thus our results may not be generalizable to other populations. Second, we only observe prescriptions that were submitted as claims to Medicare. If patients paid cash for prescriptions, they would not have been considered in our analysis. Third, our data are administrative, and we are unable to do chart reviews (eg, to examine the exact indication for the antimicrobial prescription). Finally, although we include many covariates in these models, some degree of bias because of omitted variables may persist.

Despite our limitations, we show that much of the perceived risk of adverse cardiac events associated with the use of antimicrobials is most likely associated with patient characteristics and conditions related to the prescribing of antimicrobials rather than the direct effects of antimicrobials themselves, especially for fluoroquinolones. After aggressive adjustment for a wide range of demographic and patient characteristics, there still appears to be some cardiac‐associated risk of using macrolides, highlighting the importance of appropriate antimicrobial prescribing. Nevertheless, macrolides are effective antimicrobials to treat serious respiratory tract infections and thus play an important role in the treatment of community‐acquired pneumonia.^18^ Thus, the modest potential risks attributable to these antimicrobials must be weighed against the drugs’ considerable and immediate benefits.

## Sources of Funding

This work was supported by the National Heart, Lung, and Blood Institute at the National Institutes of Health (grant K25HL122305 to Polgreen and K08HL122527 to Girotra) and the University of Iowa Health Venture's Signal Center (Polgreen).

## Disclosures

None.
